# Application of Bayesian network structure learning to identify causal variant SNPs from resequencing data

**DOI:** 10.1186/1753-6561-5-S9-S109

**Published:** 2011-11-29

**Authors:** Christopher E Schlosberg, Tae-Hwi Schwantes-An, Weimin Duan, Nancy L Saccone

**Affiliations:** 1 Division of Biology and Biomedical Sciences, Washington University School of Medicine, 660 South Euclid Avenue, Box 8226, St. Louis, MO 63110, USA; 2 Department of Genetics, Washington University School of Medicine, 4566 Scott Avenue, Box 8232, St. Louis, MO 63110, USA

## Abstract

Using single-nucleotide polymorphism (SNP) genotypes from the 1000 Genomes Project pilot3 data provided for Genetic Analysis Workshop 17 (GAW17), we applied Bayesian network structure learning (BNSL) to identify potential causal SNPs associated with the Affected phenotype. We focus on the setting in which target genes that harbor causal variants have already been chosen for resequencing; the goal was to detect true causal SNPs from among the measured variants in these genes. Examining all available SNPs in the known causal genes, BNSL produced a Bayesian network from which subsets of SNPs connected to the Affected outcome were identified and measured for statistical significance using the hypergeometric distribution. The exploratory phase of analysis for pooled replicates sometimes identified a set of involved SNPs that contained more true causal SNPs than expected by chance in the Asian population. Analyses of single replicates gave inconsistent results. No nominally significant results were found in analyses of African or European populations. Overall, the method was not able to identify sets of involved SNPs that included a higher proportion of true causal SNPs than expected by chance alone. We conclude that this method, as currently applied, is not effective for identifying causal SNPs that follow the simulation model for the GAW17 data set, which includes many rare causal SNPs.

## Background

With ongoing advances in technology, it is now possible to follow up regions of genetic linkage or association with high-throughput next-generation sequencing, which can identify novel variants, both common and rare, to be tested for association with the disease under study. The analysis of rare variants for association with disease is challenging because of the low power to determine statistically significant effects. Methods that involve collapsing or combining alleles for association testing are now popular and effective.

Our goal was to apply Bayesian network structure learning (BNSL) as a possible approach for detecting causal variants when target regions or genes have already been selected, for example, through a prior genome-wide association study. BNSL has been a successful data analysis tool in many other areas of biology, such as cell signaling pathways, systems biology, genetic data analysis, and prediction-based classification of disease [1-5]. These models create networks that extract pronounced features from the data and attempt to minimize bias from overfitting or sampling error [5]. From these examples, we hypothesized that subsets of features based on DNA sequence variation could reliably inform about the affected status in a given population and could be identified through BNSL.

## Methods

### Overall rationale and design

We are interested in the situation in which target genes or regions of interest have already been chosen (e.g., based on results from a genome-wide association study on the same or different samples) and are being followed up with resequencing to catalog and test new variants that may be causal. Even in the ideal scenario, in which all the targeted regions truly harbor causal variants, it is still challenging to identify the underlying true causal variants. We therefore requested the Genetic Analysis Workshop 17 (GAW17) answers for the purpose of narrowing our examination to genes harboring true causal variants. We focused our analyses on the 36 true genes, which contain 533 single-nucleotide polymorphisms (SNPs). Our goal was to identify causal SNPs from among all the typed SNPs in these regions. Rather than expecting to identify all causal SNPs, we sought to identify a subset of variants in which the proportion of true causal variants would be enriched compared to what would be expected by chance. The rationale is that a method that can identify a subset of SNPs that is significantly enriched for true signals should be useful for directing functional experiments and other follow-up.

We performed our analyses in two phases. The first phase was an exploratory phase in which we carried out a series of analyses under different conditions to try to determine approaches that would work well in the first 10 replicates of the GAW17 data (singly and replicates 1–10 combined). We varied several parameters and conditions, including class of learning algorithm (constraint vs. scoring methods), scoring function, number of restarts for hill-climbing algorithm, and number of perturbations of directional arcs in the graph. Even if we obtained nominally significant results in the exploratory phase, we expected that correcting for the number of tests would result in no experiment-wide significant findings. Therefore we attempted to choose the best-performing approach from the exploratory phase and applied it to the combined replicates 11–20 and also to the combined replicates 1–20. As an additional replication run, we evaluated performance in the combined replicates 21–40. We did not examine other splits or combinations of the replicates.

### Data sets

The GAW17 data set consists of 200 replicates, each containing exon sequencing data for seven distinct populations [6]. To increase sample size, we pooled populations of similar geographic and ethnic origin to obtain European ancestry, Asian ancestry, and African ancestry samples. Eigenstrat analyses [7] carried out by colleagues indicated that each of these three pooled samples clustered together well [8], with the Asian and European subpopulations clustering more tightly than the African subpopulations.

When analyzing each subpopulation, we excluded SNPs that were nonpolymorphic in that subpopulation. Table 1 summarizes the number of polymorphic SNPs per subpopulation in the 36 causal genes included in each component of our analysis and the number of polymorphic causal SNPs per subpopulation. The proportion of causal SNPs in each polymorphic, typed group is needed to compute the cumulative probability of detected SNPs, as described later.

**Table 1:** Polymorphic SNPs genotyped in the three analyzed sample subsets

Ancestry	Number of all polymorphic SNPs	Number of all polymorphic causal SNPs
African	260	59
Asian	280	87
European	194	56

### Bayesian networks

A Bayesian network (BN) is a directed acyclic graph in which each node contains quantitative probability information. Each node represents a random variable; in our application these random variables correspond to SNPs in the subpopulation under analysis and the variables Smoke, Sex, and Age. Smoke and Sex are discrete variables; we used the quantitative values of Age to create quartile bin values for analysis. The BN can be thought of as a set of directed edges that connect a pair of nodes. If an edge is connected from *X* to *Y*, where *X* and *Y* represent random variables, then *X* is said to be the parent of *Y* and *Y* is said to be the child of *X*. A BN consists of not only the network but also the conditional probability tables of the random variables, where the directed edges represent conditional dependencies between the nodes. Inferring the parameters of a BN from data relies on maximizing the likelihood that the observed data came from the model. To establish the topology of the network, the analyst can either specify the conditional independence relations that would exist or estimate statistics from the data to determine directional conditions of the nodes [9]. Details of this method can be found in [1,10,11].

### Bayesian network structure learning

When the BN topology is unknown, the structure and the parameters of the random variables can be simultaneously inferred from the data. In this analysis, the BNs were learned using the freely available, open source CRAN package bnlearn [12], and they were visualized using the “dot” component of the Mac OS X edition of the freely available graph visualization software Graphviz [13]. We used a score-based learning algorithm, which is a heuristic optimization algorithm that ranks the network structures according to information gained from perturbing directed edges between nodes. We applied the log-likelihood as the score statistic to compare each of the BNs found from the hill climbing algorithm with random restarts because, for this data set, it produced the most connected graphs relative to other discrete case scoring statistics available in bnlearn, such as the Akaike information criterion, the Bayesian information criterion, the Bayesian Dirichlet equivalent score, and the Dirichlet posterior density.

### Hypergeometric distribution

A method that is able to identify a subset of SNPs that is significantly enriched for true signals would be useful for directing functional experiments and other follow-up. Therefore we used BNSL to identify a subset of SNPs closely connected with the outcome variable Affected and then determined whether this subset contained more causal SNPs than expected by chance using the hypergeometric distribution. The set selection of the possible causal SNPs within the full BN is analogous to describing the number of successes in a sequence of *n* draws from a finite population without replacement. The following equation describes the probability mass function of the hypergeometric distribution at a specific value *k*:(1)

where *N* is the total number of SNPs analyzed by the BN, *n* is the total number of nodes (selected SNPs) in the specified subset, *m* is the total number of SNPs that are true variants (successes), and *k* is the number of SNPs among the *n* selected SNPs that are true variants (successes). The cumulative probability of selecting the specified number of causal SNPs in the selected subset is obtained by summing the probability mass functions inclusive through the maximum subset size:(2)

We use this formula to quantify the probability that out of *n* SNPs that might be identified in BNSL, we would observe *k* or more causal variants by chance.

### Defining the identified set of signal SNPs

Once BNSL is complete, there are several ways to choose a subset of nodes intended to identify causal SNPs. We chose to focus on the variables (SNPs) that were descendants of the variable Affected (i.e., the subtree rooted at Affected in the BN) and called this subset the descendants of Affected (DA). The rationale for selecting the DA subset was motivated by a study by Ramoni et al. [2], which used a BN to describe the joint associations of SNPs, demographic factors, and a nicotine dependence phenotype. In their resulting BN, a number of statistically associated and biologically relevant SNPs were located in the descendants of the nicotine phenotype; therefore we hypothesized that the DA subset could be enriched for true signals related to our Affected phenotype.

We also considered, as an alternative, the Markov blanket of Affected (MBA) subset, which is defined as the parents, children, and shared parents of the children of Affected and which we later found had been proposed previously [4,5]. In those studies, Rodin and colleagues applied BNSL to create a BN describing the joint associations of SNPs, other factors, and plasma apoE levels. The Markov blanket was used to select predictors of phenotype because, given the Markov blanket, the target variable is independent of all other nodes in the BN.

By definition, the intersection of the DA and MBA subsets of the variable Affected consists of the children of Affected (CA) subset, that is, the immediate DA subset. SNPs in the CA subset are therefore identified by both the DA and the MBA subsets; thus we also examined the CA subset for its ability to identify causal SNPs.

**Figure 1:**
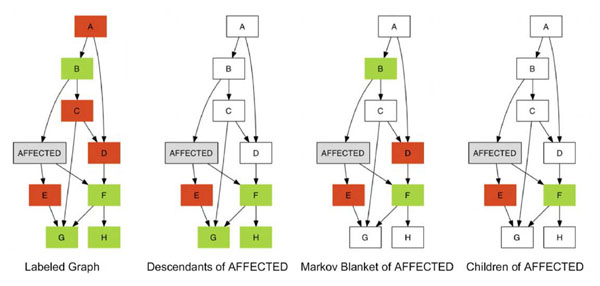
**Subset paradigms applied to identify candidate causal SNPs.** Green represents causal SNPs, and red represents noncausal SNPs. The colored portions of the three right-hand panels show the descendants of Affected (DA), the Markov blanket of Affected (MBA), and the children of Affected (CA), respectively.

Figure 1 shows an example schematic graph, where the nodes A–H represent SNPs, green represents causal SNPs, and red represents noncausal SNPs. In the three right-hand panels, the colored portions indicate the DA, MBA, and CA subsets, respectively.

### Comparison with logistic regression

We also used PLINK [14] to analyze the polymorphic typed SNPs in the target genes using logistic regression with Age and Sex as covariates in the pooled replicate sample (replicates 1–10) from BNSL. To compare the BN results to logistic regression results, we selected the top SNPs with a Bonferroni-corrected *p*-value less than 1.785 × 10^−4^ as the identified set and calculated performance using the hypergeometric distribution. We also recorded the PLINK results found for the DA subset.

## Results

### BNSL results

Table 2 summarizes the primary results, without Bonferroni correction, from the described BNSL subset selection methods, grouped by subpopulation. During preliminary analysis, the Asian subpopulation produced the highest proportion of true causal SNPs among the selected SNPs compared to the other subpopulations studied and was therefore used during the exploratory phase to discover the most appropriate restart numbers, permutation counts, BN learning method, and scoring functions.

**Table 2:** Primary results performed with log-likelihood scoring function on hill-climbing algorithm with 1,000 random restarts and 2,400 directional perturbations per score

Ancestry	Replicate	Descendants of Affected (DA)		Markov blanket of Affected (MBA)		Children of Affected (DA ∩ MBA)	
		
		Number of causal SNPs/number of SNPs	*P*(*X* ≥ *k*)	Number of causal SNPs/number of SNPs	*P*(*X* ≥ *k*)	Number of causal SNPs/number of SNPs	*P*(*X* ≥ *k*)
Asian	1–10	12/25	**0.0485**	11/45	0.891	2/3	0.228
	11–20	2/7	0.695	9/36	0.850	0/2	1
	1–20	69/182	**0.000467**	15/55	0.798	2/7	0.695
	21–40	15/69	0.983	24/63	0.113	2/4	0.367
	1	77/237	0.152	63/221	0.973	16/54	0.658
	2	75/235	0.305	55/221	0.999	17/64	0.851
	3	74/217	**0.0278**	66/190	**0.0355**	22/43	**0.00227**
	4	76/227	**0.0479**	55/190	0.894	11/42	0.821
	5	76/240	0.371	66/218	0.758	25/68	0.155
	6	74/224	0.102	55/191	0.909	13/45	0.694
	7	67/203	0.160	60/202	0.826	16/55	0.693
	8	65/212	0.663	66/226	0.937	14/61	0.958
	9	73/230	0.368	67/226	0.887	24/86	0.816
	10	65/204	0.376	59/220	0.998	15/57	0.848
European	1–10	34/105	0.155	11/46	0.849	1/6	0.874
	11–20	6/11	**0.0604**	6/22	0.655	2/2	**0.0822**
	1–20	0/1	1	2/16	0.972	0/1	0.711
	21–40	35/107	0.124	9/34	0.703	3/4	**0.0732**
African	1–10	10/60	0.929	9/47	0.795	0/3	1
	11–20	22/99	0.613	10/44	0.566	0/4	1
	1–20	7/34	0.695	11/53	0.707	0/4	1
	21–40	2/2	**0.0508**	3/17	0.786	1/1	0.226

The analysis is for all typed, polymorphic SNPs in the target gene set. Probabilities less than 0.10 are shown in bold.

Of the initial analyses of pooled replicates 1–10, only analysis of the Asian subpopulation using the DA subset showed a nominally significant *p*-value (12 true SNPs out of 25 in the DA subset; *p* = 0.0485). We then performed additional analyses focused on the Asian subpopulation samples. We analyzed the individual replicates, 1 through 10, to see whether consistent results were observed across replicates; only two replicates had *p* < 0.05, suggesting that a single replicate was too small a sample to obtain robust results. We therefore attempted to replicate the performance observed from the pooled replicates 1–10 in the combined next 10 replicates. However, in the analysis of Asian samples in pooled replicates 11–20, the DA subset did not identify significantly more true signals than expected by chance (*p* = 0.695). Follow-up analyses of replicates 1–20 did improve the significance to *p* = 0.000467 in this larger sample. However, attempted replication in the same-size, separate sample of pooled replicates 21–40 did not result in more true signals than expected by chance (15 true SNPs out of 69 in the DA subset; *p* = 0.983). Across the Asian subpopulation analyses, the only time all three subset methods (DA, MBA, and CA) resulted in *p* < 0.05 was in the analysis of individual replicate 3.

Although the Asian subpopulation was the focus of our analyses based on results from the exploratory runs, we also analyzed the African and European samples in pooled replicates 1–10, 11–21, 1–21, and 21–40. No nominally significant results were found (Table 2).

### Comparison with logistic regression results

In PLINK analyses of the Asian subpopulation, in pooled replicates 1–10, 6 out of 13 causal SNPs were found with a *p*-value less than 1.785 × 10^−4^, which produced a hypergeometric probability of *P*(*X* ≥ *k*) = 0.183. We also found that logistic regression did not detect any of the 25 SNPs found as the DA subset from BNSL analysis of this pooled sample; that is, the SNPs that were the DA subset in BNSL had *p*-values greater than the cutoff value of 1.785 × 10^−4^.

## Discussion and conclusions

We applied BNSL to all available polymorphic SNPs in genes that harbor true causal variants. We found that performing BNSL with the hill-climbing algorithm with 1,000 random restarts, 2,400 perturbations, and the log-likelihood scoring function provided the most densely connected BNs while keeping computation time reasonably low. Our analyses focused on pooled replicates; analyses of single replicates did not perform well, possibly because of the small sample size and corresponding low power. A caveat to our analyses of pooled replicates is that exact genotypes of individuals are repeated across data sets, which may have unrealistic effects on the results.

We examined three methods for identifying a subset of SNPs closely related to the disease outcome (Affected variable): the DA, the MBA, and the CA. The DA subset can be thought of as identifying SNPs connected to the disease by means of a directed path from Affected, whereas the MBA subset recognizes more proximally related SNPs such that after conditioning on the MBA subset, the remaining variables are conditionally independent of Affected. The CA subset selects the intersection of the DA and MBA subsets. During the course of our analyses, we discovered that the Markov blanket had previously been used to identify involved SNPs in an analysis of plasma lipid levels [4,5]. In our application to the GAW17 data, using the DA subset in most cases resulted in a lower *p*-value than using the MBA subset. The CA subset, which consists of the SNPs identified by both the DA and MBA subsets and thus is a consensus approach, did not identify a higher proportion of true SNPs than expected by chance and did not outperform the DA or MBA subsets. The ability of a given subset (DA, MBA, or CA) to find a greater proportion of causal SNPs reflects particular patterns of association in the analyzed data set and may not represent a general ability to perform better in any given data set.

With the DA subset, pooling the first 10 replicates gave a corresponding *p*-value of 0.0485 in the Asian subpopulation. We then applied this approach to the pooled Asian samples from replicates 11–20. We found that the *p*-value increased to 0.695, which was not consistent with the results from the combined replicates 1–10. However, the combined set of replicate sets 1–20 in the Asian samples produced a *p*-value of 0.000467. This increase in significance for replicates 1–20 pooled, compared to constituent pools that are less significant (1–10 pooled) or even nonsignificant (11–20 pooled), possibly represents the ability of BNSL to extract pronounced features from the larger data set, even if specific relationships might not be similar in subsets, because of its ability to learn from latent information in the data [11]. However, analyses of the other subpopulations (Europeans, Africans) did not indicate even nominally significant results, with suggestive results (*p* < 0.1) only in the European pooled replicates 11–20 and 21–40 and the African pooled replicates 21–40.

Analyses of larger samples (pooled replicates) tended to identify smaller SNP sets by either the MBA or the DA subset, compared to analyses of single replicates, which selected many more SNPs. For example, the MBA analysis of the Asian subpopulation included 190 to 226 SNPs in single replicate analyses, versus 45, 36, 55, and 63 SNPs in the MBA analysis of the pools 1–10, 11–20, 1–20, and 21–40, respectively. This may in part reflect the unusual duplication of genotypes across replicates, which is accompanied by varying phenotypic values, possibly resulting in fewer SNPs that would show consistent effects in the pooled data set.

We evaluated the performance of these BNSL methods by assessing whether the selected subset of SNPs was enriched for true causal SNPs compared to what would be expected by chance. Even for these few runs in the Asian subpopulation, in which nominally significant enrichment occurred (hypergeometric *p* ≤ 0.05), the proportion of true positives within these subsets ranged from 0.335 to 0.512. Although these values are higher than the baseline true positive rate of 0.311 in the Asian subpopulation (87 true causal SNPs among all 280 polymorphic SNPs), these specific selected subsets do contain many false positives. Thus it is important to note that in an application to real data, the baseline rate of true positives may be low, so enrichment may still correspond to a high false-positive rate in absolute terms.

We considered the possibility that our analyses might be identifying SNPs correlated with causal SNPs rather than the causal SNPs themselves. However, the poor performance of BNSL as applied to these data does not appear to be due to a tendency to detect *r*^2^ proxies instead of actual causal SNPs. Our linkage disequilibrium calculations show that the causal SNPs are not highly correlated with other SNPs (with one exception of a single SNP pair in the European subsample). The low *r*^2^ is consistent with the fact that so many causal SNPs have low allele frequencies. Note that *r*^2^, rather than *D*′, is the relevant linkage disequilibrium measure in this context. High |*D*′| can and does occur with rare causal SNPs; in particular, for an allele that occurs just once in one chromosome in the data set, |*D*′| is necessarily 1 with every other SNP because of the obligate zero cell in the 2 × 2 haplotype table. However, if *r*^2^ is not high, then that SNP in linkage disequilibrium is not a good predictor of allele status at the rare causal SNP.

The comparison of BNSL with the logistic regression results shows that when using each method to select a subset of SNPs in the Asian subpopulation pooled replicates 1–10, the SNPs found by logistic regression and those found by BNSL tend to have little overlap. In particular, many DA subsets have extremely high *p*-values in the logistic regression analysis. Some SNPs that had low *p*-values in the logistic regression analysis, such as C13S523 (8.60 × 10^−25^) and C13S522 (1.68 × 10^−15^), showed up as parents of Affected and were thus in the MBA subset but not the DA subset. Either way, both the traditional logistic regression analysis and BNSL methods did not demonstrate an ability to identify more true causal variants than expected by chance.

Overall, the BNSL method, as currently applied, was not able to identify sets of SNPs that included more true signals than would have been selected by chance in this simulated GAW17 data set. The difficulty may come from the challenging underlying simulation model, which involved many rare causal SNPs [6]. BNSL relies on joint and conditional probability tables, for which only limited information gain may be available from rare causal SNPs that affect a common trait. It remains possible that the method may be more effective in other data sets that differ from the GAW17 data set.

## Competing interests

The authors declare that they have no competing interests.

## Authors’ contributions

CES participated in the conception and design of the study, provided relevant programming, and drafted the manuscript. THSA provided support in the design of the study and performed the PLINK analysis. WD participated in the design of the study. NLS conceived of the study and participated in its design and coordination and helped to draft the manuscript. All authors read and approved the final manuscript.
